# The Role of Fc Gamma Receptors in Broad Protection against Influenza Viruses

**DOI:** 10.3390/vaccines6030036

**Published:** 2018-06-29

**Authors:** Natalie K. Thulin, Taia T. Wang

**Affiliations:** 1 Department of Medicine, Division of Infectious Diseases, Stanford University School of Medicine, Stanford, CA 94305, USA; nthulin@stanford.edu; 2 Chan Zuckerberg Biohub, San Francisco, CA 94158, USA

**Keywords:** universal influenza vaccine, sialylation, Fc glycan, afucosylation, Fc receptors

## Abstract

Recent studies have revealed multiple roles for Fc gamma receptors (FcγRs) in broad immunity against influenza viruses. Activating FcγR pathways can be harnessed to confer protection mediated by non-neutralizing anti-HA IgGs and to increase the potency of broadly neutralizing anti-HA IgGs and of anti-NA IgGs. Separate FcγR pathways can be targeted to enhance the breadth of antibody responses elicited by seasonal influenza virus vaccines. Here, we review the current understanding of FcγR pathways in broad influenza immunity and suggest mechanisms to bypass FcγR signaling heterogeneity among people that arises from distinctions in structural repertoires of IgG Fc domains.

## 1. Introduction

Globally, influenza viruses cause three to five million cases of severe illness and 290,000–650,000 deaths each year [1]. In the United State alone, there were 56,000 influenza related deaths during the 2010–2011 season and an estimated 30–40 million influenza-associated illnesses [2]. At this time, the primary preventative measure against influenza infections is the seasonal vaccine which is designed to induce strain-specific, neutralizing antibody responses against the strains that are predicted to circulate in the upcoming season. When circulating strains match vaccine strains, current influenza vaccines can provide substantial herd immunity. Mismatch between the predicted and actual strains in circulation occurs occasionally however, leading to reduced vaccine effectiveness [3]. In addition to risks associated with vaccine strain mismatch, the strain-specific immunity provided by seasonal influenza vaccines, along with the cumbersome vaccine production process, leaves the population vulnerable to pandemic influenza. This was exemplified in the most recent 2009 influenza pandemic in which the peak incidence of illness in the US had already passed by the time the pandemic vaccine was formulated, mass-produced, and distributed [4]. Thus, to reduce the global impact of influenza viruses, it is critical to develop methods for influenza vaccination that provide potent and broad immunity against antigenically divergent virus strains.

Antibodies elicited by current influenza vaccines are biased in specificity towards the globular head of the hemagglutinin (HA) glycoprotein; this domain is characterized by the ability to tolerate mutations, enabling seasonal virus strains to escape neutralizing antibody responses in the population by undergoing antigenic drift. Antibodies that target conserved domains of the HA or NA are not abundantly produced after influenza infection or seasonal vaccination. Thus, strategies for generation of universal influenza virus vaccines have largely focused on eliciting immunity against conserved regions of the viral HA and neuraminidase (NA) proteins. Efficacy of these vaccines is likely to depend, in large part, on the quality of broadly reactive antibodies they elicit. Recent studies have revealed that both IgG avidity and the capacity to recruit effector cell responses through Fc–FcγR interactions impact the breadth and potency of influenza antibody responses.

### IgG Antibodies and Their Receptors

**Figure 1:**
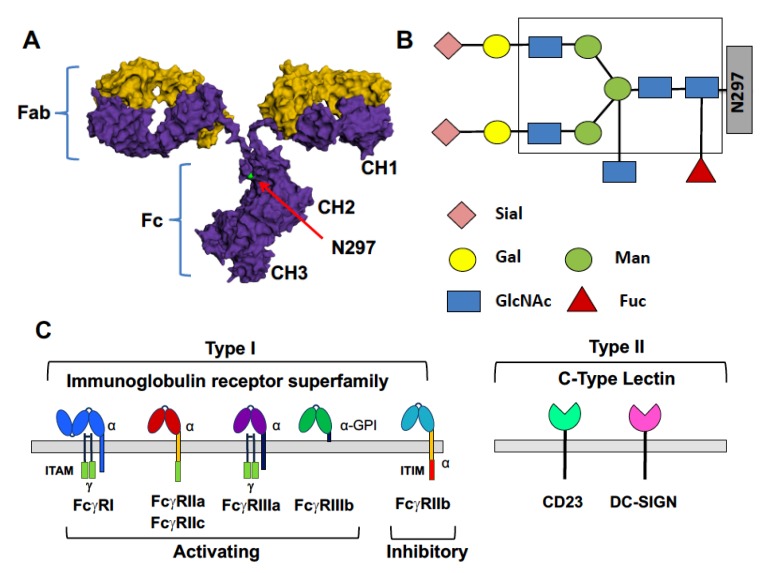
IgG antibodies and their receptors. (**A**) The structure of IgG molecules is composed of two identical heavy (purple) and light chains (yellow). Heavy and light chains combine to form Fab domains which bind antigen. The paired CH2 and CH3 domains of heavy chains form the IgG Fc domain. The structure of the Fc domain is dictated by IgG subclass and composition of the Fc glycan, located at N297 within the CH2; Fc structure, in turn, determines interactions with FcγRs; (**B**) Structure of the N297-linked Fc glycan. The composition of the core glycan is boxed; sialic acid (Sial), galactose (Gal), *N*-acetylglucosamine (GlcNAc), and fucose (Fuc) can be added to the core structure; (**C**) Type I and Type II Fc gamma receptors (FcγRs). Type I FcγRs are members of the immunoglobulin receptor superfamily; Type II FcγRs are members of the C-type lectin family.

Immunoglobulin G (IgG) antibodies have a central role in recognizing and mediating destruction of pathogens, including viruses. IgGs are Y-shaped glycoproteins composed of two identical light and heavy chains, which come together to form two distinct functional domains (Figure 1A). The Fab domains bind to antigen, conferring specificity of the IgG response. The Fc domains mediate recruitment of effector cells through interactions with the Type I and Type II Fc gamma receptors (FcγRs). Specificity of signaling through FcγRs is accomplished due to their low affinity for monomeric IgGs. This ensures that Fc-FcγR interactions occur only in the presence of antigens when immune complexes form which can engage FcγRs through avidity interactions.

Two basic types of FcγRs are now recognized. Type I FcγRs are members of the immunoglobulin superfamily and are either activating or inhibitory in nature; Type II FcγRs, in contrast, are members of the C-type lectin family and mediate different modulatory activities (Figure 1C). Five activating Type I FcγRs are expressed in humans (FcγRI, FcγRIIa, FcγRIIc, FcγRIIIa and FcγRIIIb) as well as one inhibitory FcγR (FcγRIIb). Of the Type I receptors, FcγRI has relatively high affinity for Fcs and is occupied by monomeric IgGs in vivo. The other FcγRs are lower affinity and are engaged only when multivalent immune complexes are formed. Signaling through activating Type I FcγRs results in proinflammatory mechanisms such as antibody-dependent cellular cytotoxicity (ADCC), maturation of antigen-presenting cells, enhanced antigen presentation and proinflammatory cytokine production. Activating FcγRs signal through a common immune receptor tyrosine-based activation motif (ITAM), which is present within the alpha chain of the receptor (FcγRI, FcγRIIa, FcgRIIc) or in an associated adaptor molecule, the Fc receptor gamma chain (FcγRI, FcγRIIIa). FcγRIIIb is processed as a GPI-anchored protein which lacks intracellular signaling domains [5]. Activating FcγR signaling is balanced on most cells of the immune system by signaling through the inhibitory FcγRIIb, with the level of expression of these receptors impacting the activation threshold of the cell. FcγRIIb signals through an intracellular immune receptor tyrosine-based inhibition motif (ITIM) present within the receptors alpha chain. Balancing inhibitory signaling through FcγRIIb is essential for immune homeostasis, including maintenance of immune tolerance and production of high affinity antibody responses [6,7,8]. In addition to the canonical Type I FcγRs, there are two known Type II FcγRs, DC-SIGN and CD23. These receptors have low affinity for monomeric IgGs and, as with the low affinity Type I FcγRs, are engaged only in the presence of immune complexes. Type II FcγRs transduce distinct modulatory activities, discussed below [9].

Which FcγRs are engaged by immune complexes is determined, in large part, by the structure of the Fc domains within the complexes. The primary determinant of Fc structure is IgG subclass. Humans produce four IgG subclasses (IgG1–IgG4), each having a different amino acid sequence. Each subclass has a different binding capacity for activating and inhibitory Type I FcγRs which can be expressed as a ratio of binding affinities for activating to inhibitory receptors (A/I ratio) [10]. IgG1 and IgG3 have the highest A/I ratios while IgG2 has the lowest A/I ratio. The composition of an *N*-linked, biantennary glycan located within the CH2 domain of the Fc is the second determinant of Fc structure and thus of Fc-FcγR binding affinity (Figure 1B). The core Fc glycan, composed of seven saccharide units, is required for significant immune complex-FcγR interactions. The addition of other saccharides to the core glycan can modulate the affinity of these interactions. Modifications of the core glycan include the addition of core fucose, bisecting *N*-acetylglucosamine, galactose to one or both arms of the biantennary glycan and, in the presence of galactose, terminal sialic acids may be present. Glycan modifications that confer well characterized biological activities are fucosylation and sialylation of IgG1 antibodies. A core-fucose residue is present on the majority of Fc glycoforms and, when present on the IgG1 Fc, imparts reduced affinity of the Fc, relative to the afucosylated glycoforms, for the activating FcγRIIIa [11,12]. Absence of core fucose stabilizes the Fc-FcγRIIIa interaction through an unusual carbohydrate-carbohydrate interaction, resulting in enhanced activating signaling and effector functions [13,14]. An example of this activity is observed in follicular lymphoma patients who had improved outcomes if they received an anti-CD20 mAb that is engineered for enhanced FcγRIIIa binding [15,16]. Fucosylation and sialylation of the IgG1 Fc glycan are the determinants of binding to Type II FcγRs. Core fucosylation and sialylation results in destabilization of the Fc, enabling sampling of a ‘closed’ conformation that can engage Type II FcγRs [9]. One well characterized activity associated with Fc glycan sialylation is anti-inflammatory signaling mediated by the Type II FcγR DC-SIGN. This is observed in patients with acute inflammatory diseases who can be treated with high doses of intravenous immunoglobulin (IVIg) which is purified IgG that has been pooled from thousands of donors. Treatment of IVIg to remove sialic acids abrogates its anti-inflammatory activity while enrichment for sialylation increases anti-inflammatory activity [17]. Elegant studies reviewed elsewhere have defined how sialylation of Fc glycans enables transduction of inhibitory/anti-inflammatory signaling [11,17,18,19,20]. In addition to anti-inflammatory activity, sialylated Fc glycans act through the Type II FcγR CD23 in affinity maturation of B cell responses [8,21]. This will be discussed in some detail below.

Interestingly, recent studies show that heterogeneity in human immunity can arise from differences that exists among individuals in the distribution of both IgG subclasses and Fc glycoforms [8,22]. Differences in structural repertoires of Fc domains are present in health and can also be a consequence of disease. For example, the abundance of sialylated Fc glycoforms on IgGs elicited by the seasonal influenza vaccine in healthy adults impacts maturation of the antibody response and is a determinant of vaccine efficacy (discussed below) [8]. An example of a disease impacting Fc domain repertoires was recently shown in the context of acute dengue infections [22]. This study showed that some individuals respond to dengue infection by producing IgGs that are skewed in distribution towards the IgG1 subclass and modification with afucosylated Fc glycoforms. This activating Fc domain repertoire contributed to symptoms of severe dengue disease [22]. Overall, basal Fc domain repertoires are clearly distinct among individuals and this drives diversity in human immunity. Much remains to be learned about mechanisms contributing to the regulation of Fc domain structure.

## 2. The Role of FcγRs in Broad Protection against Influenza Viruses

### 2.1. Influenza Infections

Neutralizing antibodies (nAb) against influenza viruses bind the hemagglutinin (HA) glycoprotein, which is responsible for mediating viral entry into host cells. These nAbs generally work by blocking viral attachment to host cells and can be measured using the classic hemagglutination inhibition (HI) assay which detects antibodies that prevent association of the HA with its sialic acid receptor on host cells. Anti-HA IgGs that are HI+ bind to the globular head of the HA and are, with rare exceptions, strain-specific and highly potent in vivo. In vivo protection mediated by HI+ nAbs is not dependent on engagement of FcγRs [23,24].

In contrast to the strain-specific nAbs, a series of studies from the last decade have shown that broad, IgG-mediated protection against influenza viruses is possible [25,26,27,28,29,30,31,32]. A majority of the broadly neutralizing antibodies (bnAbs) that have been isolated bind to the stalk domain of the HA protein which is highly conserved relative to the globular head domain. Recent mechanistic studies have begun to dissect cellular pathways that can be harnessed to increase the potency of protection conferred by bnAbs. Interestingly, while strain-specific nAbs do not to require FcγR interactions for potent protective activity in vivo, expression of bnAbs as IgG subclasses with high A/I ratios enhanced the potency of their protective activity by several fold. This demonstrates a dependence on activating FcγRs for their activity in vivo [24]. This degree of enhanced potency could translate to additional months of protective activity in the context of passive transfer IgGs. Perhaps even more striking, anti-HA mAbs that bind, but that do not have neutralizing activity, could also protect in vivo if they were expressed as an IgG subclass with a high A/I ratio [23]. This finding indicates that high A/I ratio IgG subclass (IgG1 or IgG3), together with broad reactivity of the Fab domain (rather than broad in vitro neutralizing activity), may predict breadth of protective activity in vivo. In addition to anti-HA IgGs, the protective activity of anti-NA IgGs in vivo also depended on the ability to engage activating FcγRs [23]. Careful cellular studies have further dissected pathways and specific mechanisms involved in broad, FcγR-mediated protection against influenza viruses [33,34,35].

**Figure 2:**
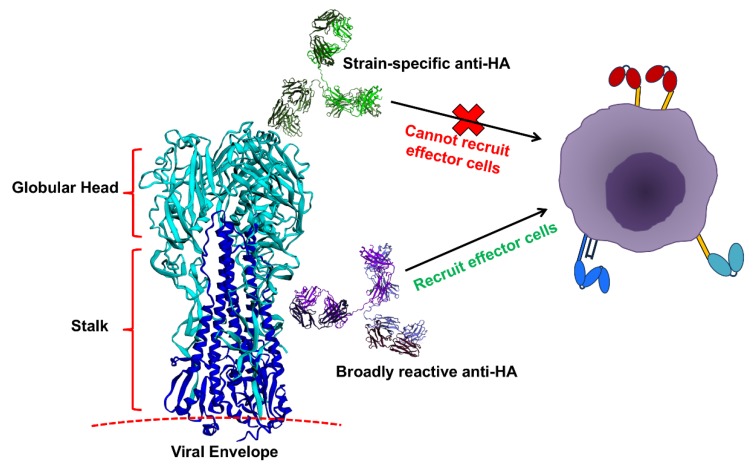
Anti-hemagglutinin (HA) specificity can dictate the capacity of IgGs to recruit effector cells. Anti-globular head antibodies that act by blocking HA-sialic acid interactions, most of which are strain-specific, do not engage FcγRs. Anti-HA IgGs that do not prevent the HA-sialic acid interactions, such as anti-stalk IgGs, can engage FcγRs, possibly due to stabilization of Fc-FcγR interactions arising from avidity interactions between HA on the infected cell with sialic acids on the effector cell. Image components not to scale.

Aside from the potency that can be achieved through harnessing FcγRs for broad protection against influenza viruses, a notable finding from these recent studies is that Fab specificity can determine whether or not IgGs can engage FcγRs. It was observed that strain-specific nAbs, which bind the globular head of the HA, could not engage activating FcγRs without engineering of the Fc for higher affinity for the receptors [24]. In contrast, broadly reactive anti-HA or anti-NA antibodies could engage FcγRs on effector cells (Figure 2) [23,24,34]. While the mechanism(s) underlying these observations are not completely understood, the existing data point to an avidity requirement for trans-engagement of FcγRs on effector cells [24,36]. The required avidity can presumably be achieved by broad-binding antibodies that do not prevent interactions between HAs on the infected cell to sialic acids of the effector cell. Thus, broadly-reactive HA stalk-reactive IgGs, for example, do not block the HA-mediated avidity interactions with the effector cell and their Fc-FcγR interactions are functionally stabilized. Regardless of mechanism, it is clear that antibodies that react with conserved influenza HA/NA epitopes can confer potent antiviral activity in vivo through Fc-FcγR mediated pathways, while strain-specific NAbs cannot engage, nor do they require FcγR interactions for potent protective activity. 

### 2.2. Influenza Vaccination

Aside from their role in broad protection of influenza viruses, FcγRs also act in the maturation of vaccine responses. This is because immune complexes form upon vaccination between vaccine antigens and existing or newly produced IgGs. Immune complexes, in turn, engage FcγRs to mediate several cellular processes involved in the maturation of high affinity antibody responses but the composition of Fc domain structures within the immune complexes that form will dictate the quality of these responses. These include: efficient transport of antigen to the germinal center, processing and presentation of antigens to T cells, and selection of B cells based on affinity of the B cell receptor (BCR) [8,37,38,39,40,41]. The role of immune complexes in maturation of influenza virus vaccine responses is particularly significant since nearly all noninfant vaccines have had exposure to influenza antigens and thus produce IgGs that react with influenza vaccine antigens.

**Figure 3:**
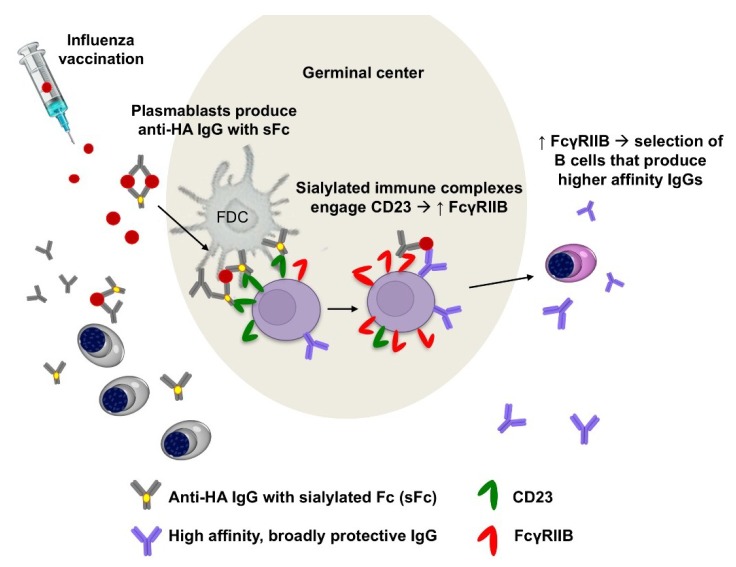
Sialylated IgG immune complexes formed during vaccination enhance affinity of the B cell repertoire. In humans, vaccination with the seasonal influenza virus vaccine triggers production of sialylated anti-HA IgGs (sialylated IgGs are likely produced by plasmambasts, data not shown). Sialylated immune complexes that are formed with vaccine antigens act through CD23 to induce upregulation of FcγRIIB on B cells. Increased expression of B cell FcγRIIb results in selection of higher affinity B cells with increased breadth of protective activity against influenza viruses [8,21].

A recent study designed to interrogate the role of FcγRs in maturation of seasonal influenza virus vaccine responses showed that vaccine-elicited IgGs undergo changes in both subclass and Fc glycoform distributions in the days and weeks following immunization [8]. This finding suggested a feedback mechanism for regulating the ontogeny of the vaccine response through interactions between vaccine-IgG immune complexes and FcγR-expressing cells. A distinct elevation in production of sialylated anti-HA IgGs following influenza vaccination led to the hypothesis that those antibodies could regulate B cells in some manner through CD23, the only Type II (sialyalted Fc-binding) FcγR expressed by B cells. To investigate this, experiments were done which found that sialylated HA immune complexes can act on B cells through CD23 to trigger upregulation of the inhibitory FcγRIIb (Figure 3). This finding was intriguing as FcγRIIb on B cells is known to play a critical role in regulating thresholds of B cell activation based on affinity of the BCR for antigen [6,42,43,44]. Subsequent in vivo experiments showed that vaccination of mice with sialylated HA immune complexes resulted in antibody responses with increased anti-HA avidity. The higher avidity anti-HA IgGs conferred increased breadth and potency of protection against multiple H1 influenza strains through enhanced antistalk immunity [8,21]. 

The finding that sialylated immune complexes elicited enhanced immunity led to additional studies aimed at determining whether the B cell CD23 pathway could be targeted to improve seasonal influenza vaccination. This work aimed to define an anti-HA monoclonal antibody, engineered at the Fc domain to engage CD23, that could be used to adjuvant existing seasonal vaccines. To this end, a bispecific monoclonal antibody was selected, comprised of the broadly reactive 19-4G05 [45] and FI6 [25], that engages both H1 and H3 subtype HA glycoproteins. This antibody was modified by sialylation of the Fc or by using a protein modification, F241A, that mimics the biological activity of sialylated Fcs [46]. Vaccination with this antibody in combination with the seasonal influenza vaccine elicited anti-HA IgGs with increased neutralization potency in vitro, increased protective potency in vivo, and increased breadth of protective activity in vivo [21]. These studies found that increasing the avidity of the anti-HA response can improve the breadth and potency of anti-influenza immunity and that harnessing the B cell CD23-FcγRIIb axis can be done to improve immunity elicited by existing seasonal influenza virus vaccines. 

While sialylation of anti-HA IgGs can modulate maturation of seasonal influenza vaccine responses, it is not yet known how Fc sialylation of anti-HA/NA IgGs may impact their protective activity during infections. Fc sialylation (in the presence of core fucosylation) can diminish affinity of the Fc for Type I FcγRs, including the activating FcγRs that mediate potent, broad protection by bnAbs in vivo (discussed in Section 2.1) [12,47]. While the diminished affinity can be measured in the lab, the function of sialylated Fc IgGs through Type I FcγRs is not necessarily impacted in vivo when avidity interactions between immune complexes and FcγRs determine cellular outcomes [48]. Thus, Fc sialyaltion may not impact the ability of bnAbs to act in vivo. Finally, it is not known how Fc sialylation may impact the pathogenesis of influenza disease by modulating inflammation. Since Fc sialylation can impart anti-inflammatory activity through a Type II FcγR-mediated pathway, the hallmark inflammatory disease associated with influenza infections might be affected by Fc sialylation of anti-influenza IgGs [7,8]. These and other important questions about the role of different anti-HA/NA Fc modifications in influenza infection and vaccination remain to be studied. 

## 3. Conclusions

Recent studies have revealed the critical roles that Type I and Type II FcγRs play in vivo in enhancing the protective potency of antibodies during influenza infection and in the ontogeny of broadly protective influenza vaccine responses. Given what has been learned about the role of activating FcγRs in mediating broad influenza immunity, an important area for future studies will be to further define how vaccine adjuvants may be used to improve the breadth of influenza vaccine responses [49,50]. We anticipate that adjuvants that can durably skew broadly reactive IgGs to subclasses with high A/I ratios will significantly improve universal influenza vaccine efficacy. Further, vaccine strategies, such as direct targeting of the B cell CD23/FcγRIIb axis to improve avidity of the antibody response, may help to bypass heterogeneity of vaccine responses that arises from differential Fc domain sialylation across the population [8].
